# Design and Performance Testing of a DNA Extraction Assay for Sensitive and Reliable Quantification of Acetic Acid Bacteria Directly in Red Wine Using Real Time PCR

**DOI:** 10.3389/fmicb.2016.00831

**Published:** 2016-06-01

**Authors:** Cédric Longin, Michèle Guilloux-Benatier, Hervé Alexandre

**Affiliations:** Equipe VAlMiS (Vin, Aliment, Microbiologie, Stress), Institut Universitaire de la Vigne et du Vin Jules Guyot, UMR Procédés Alimentaires et Microbiologiques, AgroSup Dijon – Université de BourgogneDijon, France

**Keywords:** acetic acid bacteria, red wine, microbiological internal control, DNA extraction, real time PCR

## Abstract

Although strategies exist to prevent AAB contamination, the increased interest for wines with low sulfite addition leads to greater AAB spoilage. Hence, there is a real need for a rapid, specific, sensitive, and reliable method for detecting these spoilage bacteria. All these requirements are met by real time Polymerase Chain Reaction (or quantitative PCR; qPCR). Here, we compare existing methods of isolating DNA and their adaptation to a red wine matrix. Two different protocols for isolating DNA and three PCR mix compositions were tested to select the best method. The addition of insoluble polyvinylpolypyrrolidone (PVPP) at 1% (*v*/*v*) during DNA extraction using a protocol succeeded in eliminating PCR inhibitors from red wine. We developed a bacterial internal control which was efficient in avoiding false negative results due to decreases in the efficiency of DNA isolation and/or amplification. The specificity, linearity, repeatability, and reproducibility of the method were evaluated. A standard curve was established for the enumeration of AAB inoculated into red wines. The limit of quantification in red wine was 3.7 log AAB/mL and about 2.8 log AAB/mL when the volume of the samples was increased from 1 to 10 mL. Thus, the DNA extraction method developed in this paper allows sensitive and reliable AAB quantification without underestimation thanks to the presence of an internal control. Moreover, monitoring of both the AAB population and the amount of acetic acid in ethanol medium and red wine highlighted that a minimum about 6.0 log cells/mL of AAB is needed to significantly increase the production of acetic acid leading to spoilage.

## Introduction

Acetic Acid Bacteria (AAB) species typically associated with grapes and must is *Gluconobacter oxydans* (*G. oxydans*) which prefers a sugar rich environment (Joyeux et al., 1984; Bartowsky and Henschke, 2008). AAB associated with wine are *Acetobacter aceti* (*A. aceti*) and *Acetobacter pasteurianus* (*A. pasteurianus*) which prefer ethanol as a carbon source, as does *Gluconacetobacter liquefaciens* (*Ga. liquefaciens*) (Joyeux et al., 1984; Drysdale and Fleet, 1985; Yamada et al., 1997). When these AAB are present during winemaking, aging or wine storage, they metabolize ethanol to acetaldehyde by alcohol dehydrogenase and then produce acetic acid by acetaldehyde dehydrogenase. Acetic acid is the main constituent of wine volatile acidity (Bartowsky and Henschke, 2008) and considered to be undesirable in dry wine at concentrations exceeding 0.4–0.5 g/L depending on wine type (Davis et al., 1985; Eglinton and Henschke, 1999a,b). The European regulation (CE 1308/2013) has set out limits for sale at 1.20 and 1.08 g/L acetic acid for red wines and white/rosé wines, respectively. Taking into account the ability of AAB to convert ethanol into acetic acid, these bacteria are considered as spoilage bacteria in the wine industry. Plate counting is typically used in order to quantify AAB in wine. However, culturing and enumerating AAB is challenging despite the availability of various growth media. Many studies have reported that plate counting is not appropriate for estimating AAB populations in stressful environments like wine (Sievers et al., 1992; Sokollek et al., 1998; Millet and Lonvaud-Funel, 2000; Bartowsky et al., 2003; Trcek, 2005). Indeed, this technique often underestimates AAB populations (Bartowsky and Henschke, 2008). According to Millet and Lonvaud-Funel (2000), the difficulties of isolating AAB may be due in part to the existence of a Viable But Non Culturable (VBNC) state which may result from anaerobic conditions in wine (Du Toit et al., 2005). Many techniques can be used for AAB detection like nested PCR (González et al., 2006), AAB gene (*adh*A) PCR (Trcek, 2005), PCR-Restriction Fragment Length Polymorphism (Ruiz et al., 2000), and many others, however, all these techniques are culture dependent and the VBNC state may lead to underestimating the AAB population. Independent culture quantification techniques using Denaturing Gradient Gel Electrophoresis (De Vero et al., 2006), Temperature Gradient Gel Electrophoresis (Ilabaca et al., 2008), or epifluorescence have been reported (Mesa et al., 2003; Baena-Ruano et al., 2006). These latter authors quantified AAB in vinegar fermentation using viability (i.e., measurement of cell membrane permeability) and vitality (i.e., measurement of cell enzymatic activity) dyes. This technique has a high detection limit and appreciation of fluorescence is operator dependent. Thus, qPCR techniques have been developed (Torija et al., 2010; Valera et al., 2013) to quantify AAB. The qPCR method presented by González et al. (2006) did not use any internal control and was validated with red wine inoculated with a known amount of AAB, but without any growth. However, AAB growth in wine modifies DNA extraction efficiency and qPCR reliability since polyphenols seem to be adsorbed onto the cell walls (Morata et al., 2004). In addition, problems often arise with DNA amplification due to inhibitory substances such as tannins, polysaccharides and pigments (Rossen et al., 1992; Wilson, 1997). Therefore, poor DNA isolation and/or amplification efficiency, leading to false-negative results, were observed (Tessonnière et al., 2009). The specific species quantification of AAB using TaqMan probes was also reported (Torija et al., 2010; Valera et al., 2013). However, these probes are expensive and could not be used for routine laboratory analysis to determine total AAB population. Furthermore, none of these assays used internal controls to screen for the presence of inhibitors contained in the samples, leading to unreliable quantification. The goal of the current work was to develop a qPCR technique using an internal control that provides efficient and reliable quantification of the AAB naturally present in red wine. Different DNA extraction protocols were compared in order to remove wine inhibitors and assess the sensitivity, specificity, and reproducibility of the method.

## Materials and methods

### Microorganism strains

Four different species of AAB were used: *Acetobacter aceti* DSM 3508 (DSMZ-Deutsche Sammlung von Mikroorganismen und Zellkulturen GmbH, Braunschweig, Germany), *Acetobacter pasteurianus* CECT 7582 (Colección española de cultivos tipo, Universitat de València, Edificio de Investigación, Burjassot, Spain); *Gluconobacter oxydans* DSM 7145*; Gluconacetobacter liquefaciens* CIP 103109 (Collection of Institut Pasteur, Biological Resource Center of Institut Pasteur (CRBIP), Paris, France). *Oenococcus oeni* sabo11 (a biotechnological strain isolated from a South African wine) was also used as the majority LAB present in wine. AAB were adapted to ethanol by growing them in mannitol medium supplemented with ethanol [2.5% (w/v) mannitol; 0.5% (w/v) yeast extract; 0.3% (w/v) peptone, 5% (v/v) ethanol]. *O. oeni* was adapted in FT80 medium (Cavin et al., 1988). *Escherichia coli* K12 ER2738 (available from New England Biolabs) was cultivated in LB medium (1% (*w*/*v*) peptone, 0.5% (*w*/*v*) yeast extract, 1% (*w*/*v*) NaCl). Yeasts, namely *Zygosaccharomyces bailii* MUCL 27812 (Mycothèque, de l'Université Catholique de Louvain, Louvain-la-Neuve, Belgium), *Candida vini* MUCL 27720, *Pichia membranifaciens* PMmb2000*, P*. *fermentans* PFmb2005, and *Saccharomyces cerevisiae* FERMOL-PB2023, were grown in modified YPD medium (2% (*w*/*v*) glucose; 0.5% (*w*/*v*) yeasts extract, 1% (*w*/*v*) peptone) with chloramphenicol at 0.02% (*w*/*v*) added after sterilization.

### Growth conditions

For the artificial contaminations, adapted AAB were inoculated into mannitol medium supplemented with 10% (*v*/*v*) ethanol (ethanol medium), white and red wines. Growth was ensured at 28°C. Red wine made from Pinot Noir grapes was supplied by the vineyard of the University of Burgundy [pH: 3.5; 12% (*v*/*v*) alcohol] as was white wine made from Chardonnay grapes [pH: 3.5; 12% (*v*/*v*) alcohol]. The wines were filtered through a 0.2 μm sterile membrane and dispensed into sterilized Erlenmeyer flasks.

### Enumeration of microorganisms

After AAB growth in ethanol medium and white and red wines, bacteria levels were measured by flow cytometry (FCM) in BD Accuri C6 flow cytometers with a single dye DiBAC_4_(3) [Bis-(1,3-Dibutylbarbituric Acid)Trimethine Oxonol] (ThermoFisher Scientific, Molecular Probes™, B-438) (final concentration 6 μM) in PBS buffer 1X (Biosolve, 10X concentrate Molecular biology, 162323). This compound was excited by the flow cytometer laser at 488 nm and emitted fluorescence collected by the filter 530 ± 15 nm. This dye was used for counterstaining and circumventing potential culture dependent shortcomings such as the VBNC state of wine microorganisms (Millet and Lonvaud-Funel, 2000). Three enumeration repetitions (20 μL at 34 μL/min) were performed for each sample.

### Internal control for DNA isolation and amplification

Specific EC23S primers were selected to quantify *E. coli* K12 ER2738 (Forward: 5′-CATAAGCGTCGCTGCCG-3′; Reverse: 5′-AAAGAAAGCGTAATAGCTCACTGGTC-3′) (Ludwig and Schleifer, 2000; Chern et al., 2011). A standard curve with these primers using the 23S rRNA gene sequences as target was obtained in LB medium at 10^7^ to 10^1^ cells/mL. 20 μL of internal control at 5.10^5^ *E. coli*/mL were added to each 1 mL sample prior to DNA isolation to obtain a concentration of 10^4^ *E. coli*/mL in each sample.

### DNA isolation and extraction

After AAB growth and the addition of the internal control, two DNA extraction methods were tested: the Ausubel (Ausubel et al., 1992) and Lipp methods (Lipp et al., 1999). The two methods use CTAB as Jara et al. (2008) recommend. The main differences are that the second method use chloroform to purify DNA and use a CTAB precipitation solution after cell lysis to eliminate the remaining polyphenols. Moreover, DNA was re-dissolved in 50 μL sterile deionized water for the methods. When indicated, cells were centrifuged and the pellets were resuspended into lysis buffer supplemented with PVPP at a final concentration of 1% (*w*/*v*). Each bacterium was tested in growth medium supplemented with 10% (*v*/*v*) ethanol, and white and red wines. All the experiments were performed in triplicate and repeated three times.

### AAB real-time PCR amplification (qPCR)

AAB primers used to amplify the 16S rRNA gene were selected from Valera et al. (2015). The forward primer AAB-F (5′-TGAGAGGATGATCAGCCACACT-3′) and the reverse primer AAB-R (5′-TCACACACGCGGCATTG-3′) were synthesized by Eurogentec® (France). The PCR mixture was prepared in a total volume of 25 μL with 100 nmol of each primer and 5 μL DNA extract. The amplifications were done in triplicate on a CFX90 real-time PCR system (Bio-Rad) under the following conditions: 95°C for 10 min, 40 denaturation cycles at 95°C for 15 s, and 62°C for 1 min. Then a melting curve was produced to check the presence of only one amplification fragment. To test PCR amplification quality, BSA, and PVP treatments during qPCR at 400 ng/μL and 0.5% (*w*/*v*), respectively, were performed (Tessonnière et al., 2009). A control without treatment was also performed. The PCR cycle in which fluorescence first occurred (quantification cycle: *C*_*q*_) was determined automatically using Bio-Rad CFX Manager® software after setting the regression method.

### Volatile acidity according to AAB population

The acetic acid concentration and AAB population were monitored over time in synthetic medium containing 10% (*v*/*v*) alcohol. The amount of acetic acid was measured enzymatically using a Biosentec kit (Cat. No 021) according to the manufacturer's instructions, expressed in gram per liter. The AAB population was determined throughout their growth by plating on mannitol agar (CFU/mL). The same experiment was also performed in red wine from the vineyard of the University of Burgundy but no inoculation was performed; the AAB contamination was natural. In this experiment, the AAB population was determined by both qPCR and FCM. FCM provided the total bacteria population count. LAB enumeration was performed in FT80 Petri dishes to avoid overestimating the AAB population determined by FCM. Moreover, 15 red wines were chosen randomly from different wine regions to analyze the AAB populations (qPCR and FCM). In addition, acetic acid concentration was determined.

## Results

### qPCR specificity

The specificity of the AAB primers was tested against a panel of microorganisms known to be naturally present in wine. *In silico* tests were performed on *O. oeni, Pediococcus* spp., *Lactobacillus* spp, *Zygosaccharomyces bailii, Candida vini, Pichia membranifaciens, P*. *fermentans,* and *S. cerevisiae*. AAB primers did not match with the main wine microorganisms. *In vitro* tests were performed in synthetic medium against *O. oeni* and a panel of yeasts (see above). The *C*_q_-values were the same as the negative control for all populations of *O. oeni* and yeast, thus validating the specificity of the primers. The EC23S specific primers targeting the 23S rRNA gene from *E.coli* (internal) control did not amplify DNA of the wine microorganisms tested. *E. coli* has been chosen because this microorganism is not naturally present in wine.

### Comparison of DNA isolation methods

DNA extraction for AAB quantification was performed after growth in ethanol medium, and white and red wines using either the Lipp or Ausubel methods with or without PVPP during cell lysis. In order to compare the DNA extraction methods, the *C*_q_ obtained from DNA extracted by both methods with or without PVPP for the three different media were compared for each bacterium.

As shown in Table 1A, for ethanol medium, the Lipp method without PVPP gave significantly better results, as shown by a lower *C*_q_ whatever the bacteria, except for *G. oxydans*, showing no significant difference in the results obtained by both methods. PVPP addition during DNA extraction using either the Lipp or Ausubel method did not improve qPCR efficiency for most of the bacteria tested. PVPP addition during extraction with the Ausubel method significantly improved *C*_q_ only for *A. aceti* and *A. pasteurianus*. Thus, the choice of DNA extraction method is essential.

**Table 1:** ***C*_*q*_ results according to DNA extraction (Ausubel and Lipp methods) with (+) or without (−) PVPP for *A. aceti, A. pasteurianus, G. oxydans,* and *Ga. liquefaciens* in (A) growth medium supplemented with 10% (*v*/*v*) ethanol, and (B) white, and (C) red wines**.

**(A)**					
**Methods**	**PVPP**	**Ethanol medium**			
		***A. aceti***	***A. pasteurianus***	***G. oxydans***	***Ga. liquefaciens***
Ausubel	–	32.0 ± 0.0^b^	30.5 ± 0.0^c^	21.8 ± 1.8^a^	23.0 ± 1.7^b^
	+	22.7 ± 1.7^a^	18.3 ± 0.8^b^	22.4 ± 1.7^a^	23.9 ± 0.4^b^
Lipp	–	18.5 ± 0.6^a^	14.0 ± 0.5^a^	18.6 ± 1.7^a^	20.3 ± 0.6^a^
	+	20.4 ± 2.7^a^	15.4 ± 0.6^a^	18.9 ± 0.8^a^	20.2 ± 0.6^a^
**(B)**					
**Methods**	**PVPP**	**White wine**			
		***A. aceti***	***A. pasteurianus***	***G. oxydans***	***Ga. liquefaciens***
Ausubel	–	23.9 ± 1.3^b^	21.8 ± 0.8^a^	22.4 ± 0.3^c^	23.1 ± 1.3^b^
	+	23.0 ± 2.8^ab^	22.2 ± 0.6^a^	22.2 ± 1.3^c^	21.6 ± 0.0^ab^
Lipp	–	19.8 ± 0.1^ab^	19.8 ± 0.6^a^	16.8 ± 0.6^a^	21.2 ± 0.3^a^
	+	19.6 ± 0.8^a^	19.3 ± 2.3^a^	19.4 ± 0.7^b^	20.8 ± 0.4^a^
**(C)**					
**Methods**	**PVPP**	**Red wine**			
		***A. aceti***	***A. pasteurianus***	***G. oxydans***	***Ga. liquefaciens***
Ausubel	–	26.4 ± 1.9^b^	28.4 ± 0.5^b^	25.9 ± 1.7^b^	26.2 ± 1.1^b^
	+	27.8 ± 0.8^b^	26.9 ± 0.9^b^	26.3 ± 0.8^b^	26.1 ± 0.3^b^
Lipp	–	23.2 ± 0.5^a^	12.7 ± 0.7^a^	23.8 ± 1.0^ab^	21.7 ± 0.4^a^
	+	22.4 ± 0.4^a^	11.2 ± 0.3^a^	22.1 ± 0.6^a^	21.2 ± 0.0^a^

*Values represent the C_q_ mean ± Standard Deviation (SD); n = 3; values followed by different letters within a column are statistically different at p < 0.05 (XLStat^©^)*.

For white wine (Table 1B), the effect of PVPP is species dependent, but the results confirmed that the Lipp method gave much better results.

Regarding red wines which are rich in qPCR inhibitor compounds, the Lipp method gave much better *C*_q_ compared to the Ausubel method, except for *G. oxydans*. When PVPP was added prior to DNA extraction, the *C*_*q*_-values did not improve whatever the DNA extraction method, except in a few cases (Table 1C). However, when using PVPP, the Lipp method was always better than the Ausubel method, as demonstrated by the lower *C*_q_-values. For example, in our study we obtained a *C*_q_ for *A. aceti* of 27.8 in red wine (Ausubel + PVPP). When using the Lipp method with PVPP, the *C*_q_ was equal to 22.4. Thus, using the suboptimal extraction method, *C*_q_ led to underestimating the real AAB population.

**Figure 1:**
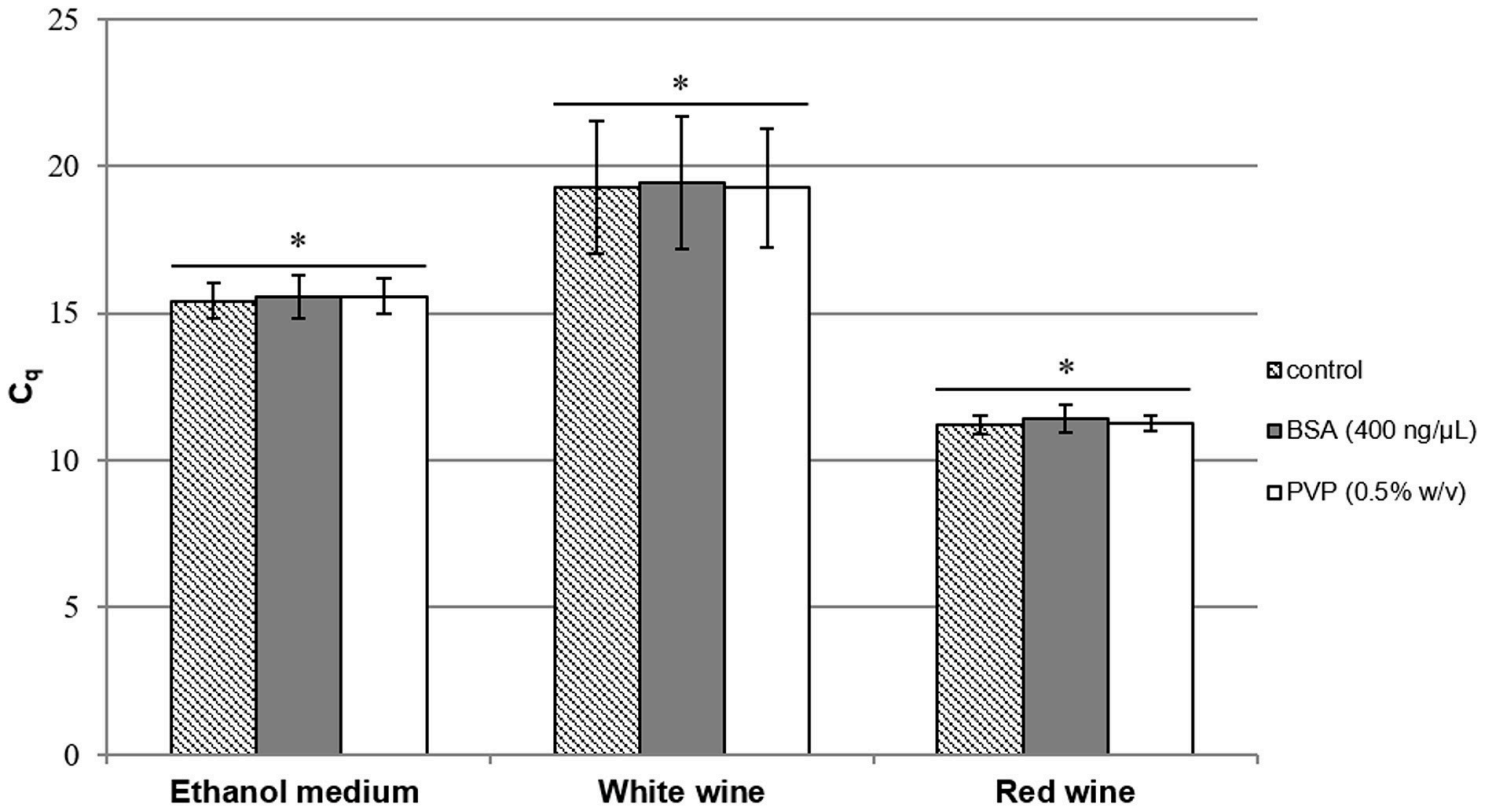
***C*_*q*_ results for *A. pasteurianus* after DNA extraction by the Lipp method in mannitol supplemented with 10% (*v*/*v*) alcohol (containing 2.4.10^6^ cells/mL), white wine (containing 2.7.10^5^ cells/mL), and red wine (containing 9.9.10^7^ cells/mL)**. Cell lysis was performed in triplicate with PVPP and BSA (400 ng/μL) (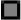) or PVP (0.5% *w*/*v*) (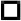), added to the qPCR mix and compared to control (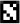). An Anova with Tukey's test was performed for each medium independently to analyze the *C*_q_ results with BSA and PVP compared to control (*p>0.05), (XLStat^©^). Error bars represent standard deviations.

Another way to improve qPCR efficiency is to add either BSA or PVP in the qPCR mix (Jiang et al., 2005; Malorny and Hoorfar, 2005). These compounds are assumed to trap inhibitors in the reaction mix. Figure 1 shows the results of PCR assays on *A. pasteurianus*. DNA was extracted using the Lipp extraction method with PVPP, and qPCR was run with or without either the addition of BSA or PVP in the qPCR mix compared to control. The addition of BSA or PVP in the qPCR mix did not lead to any improvement of the *C*_q_ obtained. No improvement of *C*_q_-values was observed using these compounds and, as no difference was highlighted in red wine with the Lipp DNA extraction method between the four species, neither BSA nor PVP were added in the mix to quantify AAB using the Lipp method in red wine. However, BSA added to *A. pasteurianus* DNA using the Ausubel method with or without PVPP in red wine significantly improved *C*_q_-values compared to control, but they were always significantly higher (less effective) than the Lipp method. The values from the Ausubel DNA extraction method with or without PVPP were higher than 11.3 and 12.8 units, respectively, compared to the values obtained with the Lipp method. These results seem to indicate the presence of PCR inhibitor compounds in DNA extract using the Ausubel method with or without PVPP.

### Use of *E. coli* ER2738 as microbiological internal control

An *E. coli* standard curve from DNA isolation at various levels between 10^1^ and 10^7^ bacteria/mL was used to evaluate qPCR efficiency (98.8%) with a correlation coefficient of 0.998. The *T*_m_ of the product had a value of 82.5 ± 0.5°C. The trend curve is: [*E*. *coli*]_concentration_(log cells∕mL) = −0.2977 × C_q_+11.581. A suspension of the strain *E. coli* K12 ER2738 was added to obtain 10^4^ cells/mL in ethanol medium (10% *v*/*v*), and white and red wines samples containing *A. aceti, A. pasteurianus, G. oxydans,* and *Ga. liquefaciens*. According to the standard curve, efficient DNA extraction and amplification with EC23S primers should give a *C*_q_-value of 25.5 ± 0.4 for 4 log cells/mL of *E. coli*. Thus, following DNA extraction performed in triplicate of each AAB in each media with both Ausubel and Lipp methods, with or without PVPP, *E. coli* was quantified.

The values in Table 2 are the averages of *E. coli* populations found after DNA extraction according to methods with or without PVPP. No significant differences (*p*>0.05) can be seen between the two techniques used for DNA extraction with or without PVPP for *E. coli* enumeration in ethanol medium and white wine. No significant cell loss was highlighted for either the Ausubel or Lipp method. However, the standard deviation of the Lipp method was lower than 0.3 log *E. coli*/mL compared to the Ausubel method for both media.

**Table 2:** **Averages of *E. coli* K12 ER2738 found after initially adding 4 log cells/mL in ethanol growth medium and white and red wines containing *A. aceti, A. pasteurianus, G. oxydans,* and *Ga. liquefaciens,* and following DNA extraction**.

**Methods**	**PVPP**	**Ethanol medium**		**White wine**		**Red wine**	
		**log *E. coli*/mL**	***p*-values**	**log *E. coli*/mL**	***p*-values**	**log *E. coli*/mL**	***p*-values**
Ausubel	−	3.4 ± 0.7^a^	*p* > 0.05	3.6 ± 0.5^a^	*p* > 0.05	1.9 ± 0.5^b^	*p* < 0.05
	+	3.5 ± 0.4^a^	*p* > 0.05	3.7 ± 0.5^a^	*p* > 0.05	1.8 ± 0.2^b^	*p* < 0.05
Lipp	−	3.9 ± 0.3^a^	*p* > 0.05	3.6 ± 0.1^a^	*p* > 0.05	3.3 ± 0.2^a^	*p* > 0.05
	+	3.6 ± 0.2^a^	*p* > 0.05	3.6 ± 0.3^a^	*p* > 0.05	3.7 ± 0.1^a^	*p* > 0.05

*The Lipp and Ausubel methods were used with (+) or without (−) PVPP during cell lysis. qPCR was done with EC23S primers*.

*An Anova representation with Tukey's test was applied to the E. coli enumeration results using as control modality of 4.0 log E. coli/mL, (XLStat^©^). Values followed by different letters within a column are statistically different at p < 0.05 (XLStat^©^)*.

In red wine, the mean values for *E. coli* concentration in the Lipp method after DNA extraction with or without PVPP were 3.7 ± 0.2 and 3.3 ± 0.2 log cells/mL, respectively, whereas Ausubel DNA extractions led to 1.8 ± 0.2 and 1.9 ± 0.5 log cells/mL, respectively, with and without PVPP. Thus, as shown in Table 2, the Lipp method with or without PVPP led to the recovery of the internal control population, which was not significantly different from the added population. Although not significant, the *C*_q_-value following Lipp DNA extraction with PVPP tended to be slightly lower in red wine compared to experiments performed without PVPP. On the basis of these results, the Lipp DNA extraction method with PVPP was used for all extractions in wine. AAB can be quantified only if *E. coli* quantification is not significantly different from the added concentration (10^4^ *E. coli*/mL), since the detection of a loss of internal standard leads to an underestimation of AAB population.

### AAB quantification with the presence of other wine microorganisms

To verify the absence of interference by other microorganisms in naturally contaminated wine, the following procedure was implemented. AAB quantifications in red wine containing 10^5^ *A. pasteurianus*/mL alone or supplemented with *O. oeni* and *B. bruxellensis* at 10^5^ cells/ml were performed. qPCR with AAB primers was performed after validating the presence of *E. coli* at 10^4^ cells/mL. The results of the samples containing *A. pasteurianus* alone or with other microorganisms (*O. oeni* and *B. bruxellensis*) were identical: 5.1 ± 0.1 and 5.1 ± 0.2 log *A. pasteurianus*/mL, respectively.

### Linearity, repeatability, and reproducibility in red wine

**Figure 2:**
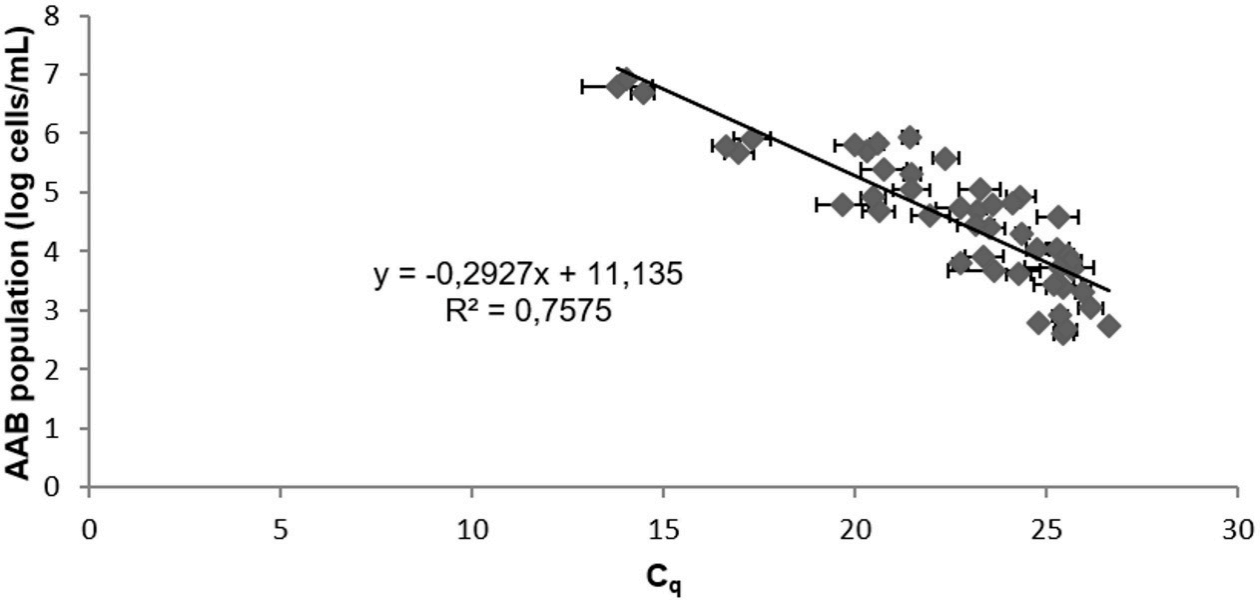
**Standard curve from 10-fold serial sample dilutions of red wines containing *A*. *aceti*, *A. pasteurianus*, *G. oxydans, Ga. liquefaciens*, and the AAB mixture**. The *C*_q_ values are the average of three individual experiments performed in triplicate. Error bars represent standard deviations.

After artificial contamination of the red wine by AAB and incubation allowing their growth, verified over time by flow cytometry, Lipp DNA extraction with PVPP was performed in triplicate with 3 repetitive independent experiments. The *T*_*m*_ of the product had a value of 82.5 ± 0.5°C. AAB quantification could be done after confirming the presence of 4 log *E. coli*/mL. Efficiency, *r*^2^, and y-intercept are presented in Table 3. No significant differences between efficiency, *r*^2^, and y-intercept following a Tukey's test between red wines, containing each AAB species or a mix of all AAB, were observed. Thus, all the data were compiled to determine the general standard curve allowing AAB quantification in red wine whatever the species present (Figure 2):

$$\begin{array}{l} {\left[AAB\right]}_{\text{concentration}}\left(log\;cells∕mL\right)\;=-0.2927\;\times \;C_{q\;+11.135}. \end{array}$$

**Table 3:** **Statistical analysis of qPCR parameters obtained from independent DNA isolation experiments performed on *A. aceti, A. pasteurianus. G. oxydans,* and *Ga. liquefaciens* after growth in red wine in triplicate and with three repetitions of the experiment over time**.

	**Efficiency**	***r*^2^**	**y-intercept**
*A. aceti*	166 ± 46^a^	0.926 ± 0.031^a^	35.2 ± 3.0^a^
*A. pasteurianus*	176 ± 37^a^	0.942 ± 0.069^a^	33.8 ± 1.7^a^
*G. oxydans*	167 ± 40^a^	0.940 ± 0.018^a^	34.4 ± 1.9^a^
*Ga. liquefaciens*	178 ± 33^a^	0.875 ± 0.098^a^	33.5 ± 1.8^a^
AAB mixture	125 ± 09^a^	0.988 ± 0.012^a^	33.6 ± 0.7^a^
*p* values	*p* > 0.05	*p* > 0.05	*p* > 0.05

*Three red wines containing a mix of AAB were also analyzed in triplicate. An Anova with Tukey's test were performed according to efficiency, r^2^, and y-intercept, (XLStat^©^). Values followed by the same letter within a column are statistically identical at p > 0.05 (XLStat^©^)*.

### Limit of quantification (LOQ) of AAB in red wine

For our study, LOQ was determined using the slope, residue standard deviation, and standard deviation of the intercept obtained from linearity validation experiments (OIV, 2005). Using the Lipp method after AAB growth in red wine, the LOQ value of 5.2.10^3^ cells/mL was obtained. These results were the mean of the three independent experiments performed in triplicate. Moreover, the sampling volume of 10 mL instead of 1 mL led to the improvement of LOQ by 0.9 ± 0.1 log cells/mL.

### Acetic acid monitoring according to AAB population

**Figure 3:**
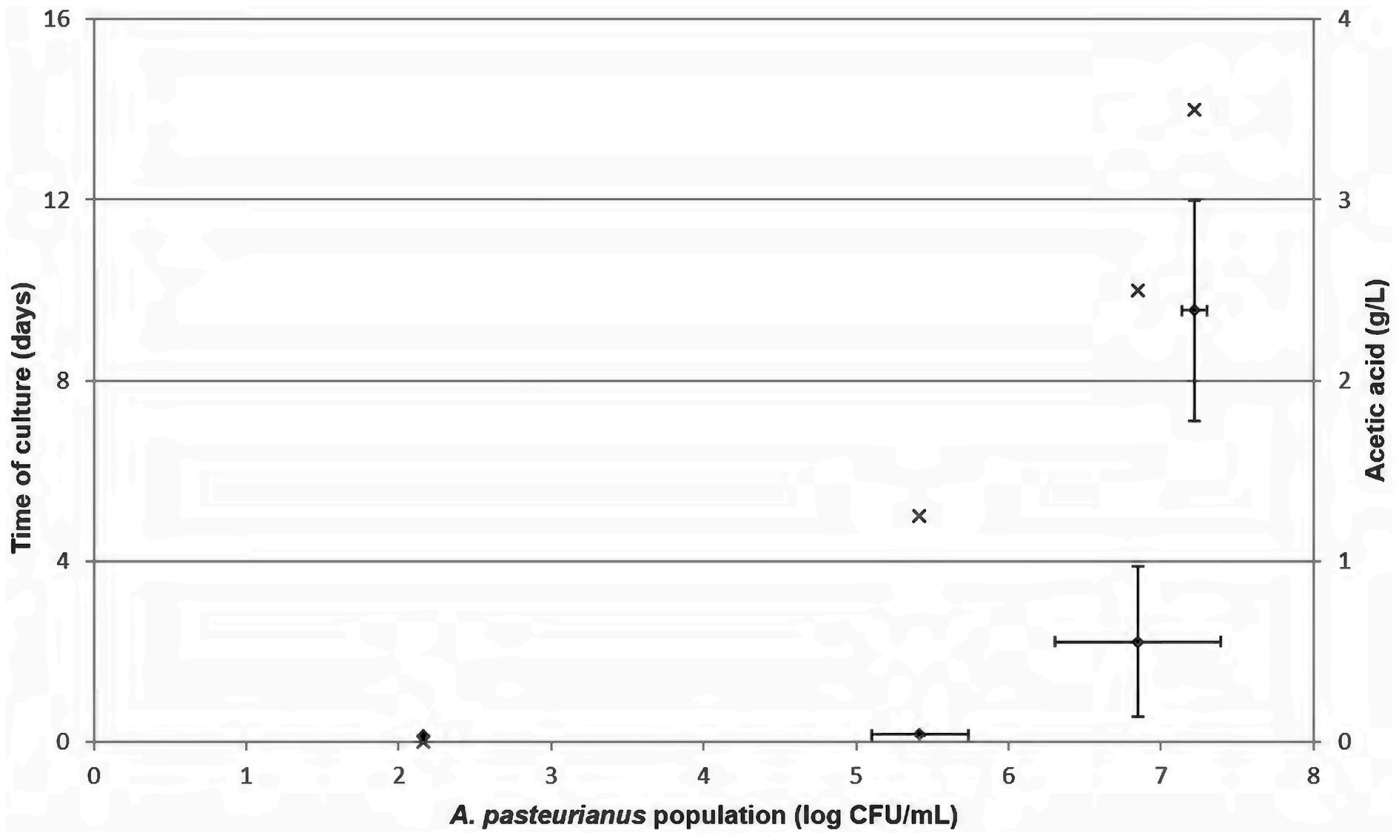
**Monitoring of *Acetobacter pasteurianus* growth in mannitol medium containing 10% (*v*/*v*) alcohol (×)**. Initial inoculation was 10^2^ cells/mL. This experiment was performed in three independent experiments. Acetic acid concentration (♦) was determined in triplicate over time. Error bars represent standard deviations.

Acetic acid concentration monitoring was performed after *A. pasteurianus* inoculation in ethanol medium [10% (*v*/*v*)] at 10^2^ cells/mL in independent experiments performed in triplicate (Figure 3). The initial amount of acetic acid was 0.04 g/L. Figure 3 shows acceptable acetic acid values of 0.04 ± 0.00 g/L for an AAB concentration from 2.2 to 5.4 log CFU/mL. The sample presented 0.52 g/L acetic acid when the *A. pasteurianus* population reached 6.9 log CFU/mL. The last analysis point has a bacterial population of 7.2 ± 0.1 log CFU/mL and shows acetic acid values from 2.39 ± 0.61 g/L. Thus, acetic acid production increased in ethanol medium and occurred when acetic acid bacteria exceeded 6 log CFU/mL. AAB growth was low between the analysis points of 10 and 14 days, indicating that culture reached a stationary phase. The high acetic acid concentration determined during this time seemed to highlight overproduction during the stationary phase of the culture, i.e., when AAB are in high concentration.

**Figure 4:**
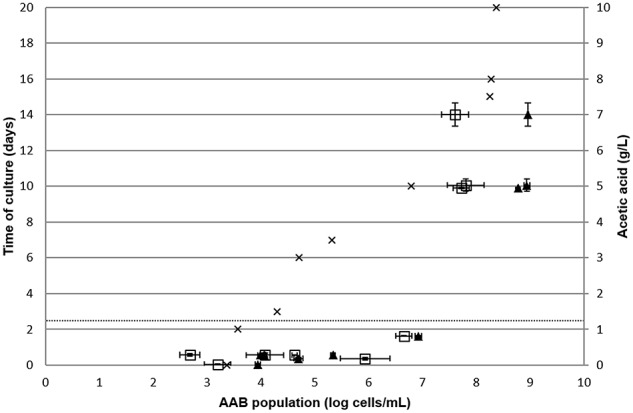
**Monitoring of natural AAB growth in red wine**. The AAB population was determined by FCM (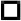) and qPCR (▴) in duplicate over time. Acetic acid concentration was determined in duplicated over time. The dotted line represents the limit of the European threshold. Error bars represent standard deviations.

To confirm these hypotheses of acetic acid production beyond acceptable limits at a population higher than 6 log AAB/mL and during the stationary phase of AAB growth, AAB growth was monitored in red wine from the vineyard of the University of Burgundy, contaminated naturally over time (Figure 4). Both the AAB population and acetic acid concentration were determined over time. The enumeration of total bacteria in the samples was performed by FCM and LAB quantification in specific medium. Neither yeasts nor LAB were detected in the wine under study, consequently only AAB were present and enumerated. Therefore, it was possible to compare the AAB concentration determined by FCM/qPCR methods and acetic acid production. Acetic acid concentration as a function of AAB population is presented in Figure 4. For the first points of analysis containing a population ranging from 2.9 to 6 log AAB/mL, quantifications by qPCR were well-correlated with quantification by FCM. The acetic acid concentrations at these analysis points did not exceed 0.29 g/L. Then, higher acetic acid concentrations were reached for higher acetic acid bacteria concentrations. The analysis point containing an AAB population of 6.8 ± 0.2 log bacteria/mL had 0.82 ± 0.01 g/L acetic acid. The last point of AAB growth monitoring in red wine presented a high AAB concentration with acetic acid levels exceeding the European limit values and an increase in the mean difference between the qPCR and FCM methods (1.2 ± 0.6 log cells/mL). Figure 4 validates acetic acid production by AAB during the stationary growth phase, thus when AAB exceeded 6 log bacteria/mL.

**Figure 5:**
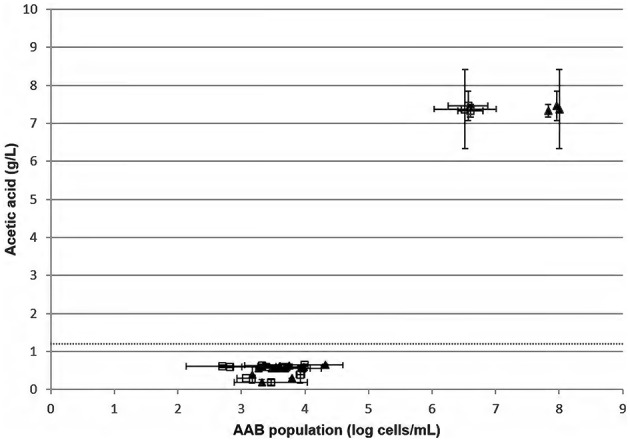
**Measure of acetic acid concentration in duplicate according to the AAB population determined by FCM (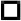) and qPCR (▴) in 15 red wines from different wine regions chosen randomly**. The dotted line represents the limit of the European threshold.

The measure of acetic acid and counting of AAB by FCM/qPCR methods were then performed on 15 red wines chosen randomly from wine regions (Figure 5). Twelve red wines had a mean amount of acetic acid at 0.52 ± 0.15 g/L. For these same samples, the median amount of acetic acid was 0.57 ± 0.15 g/L. These wines are considered as unspoiled. The mean relative difference between FCM vs. qPCR, allowing the determination of the AAB population, was 0.5 ± 0.3 log cells/mL. Three of the fifteen red wines analyzed presented a high amount of acetic acid and were thus spoiled. These wines had an AAB concentration higher than 6 log AAB/mL.

## Discussion

Cell quantification by qPCR in red wine is difficult since various qPCR inhibitors such as polyphenols and polysaccharides are abundant, thereby increasing the risk of false negative results (Demeke and Jenkins, 2009) and making the amplification of genetic material challenging. qPCR has been used to quantify AAB in wine (González et al., 2006; Andorrà et al., 2008; Torija et al., 2010; Valera et al., 2013), however, the authors of these studies did not take into account extraction efficiency or the presence of inhibitors, nor did they use any control process (cells added to the matrix). Process control can provide information on the efficiency of extraction and on the PCR procedure. The addition of a “spike” control to ensure accurate and reliable quantification is important (Fukushima et al., 2003; Stoeckel et al., 2009; van Doorn et al., 2009) and has already been reported for food analysis (Josefsen et al., 2010; Krøjgaard et al., 2011; Ishii et al., 2013), but as far as we know only once for quantifying *Brettanomyces* in wine (Tessonnière et al., 2009). In order to develop an accurate and efficient qPCR method to quantify AAB, DNA extraction procedures were first tested in order to ensure good DNA preparation. Indeed, the extraction efficiency and quality of DNA must be optimized for quantitative PCR. The selection of an appropriate DNA extraction method from those available is thus crucial (Jara et al., 2008).

### DNA extraction method

We opted for the Lipp method (Lipp et al., 1999) since this method has proven useful for many foodstuffs, especially those rich in phenolic compounds. By comparing this method with classical CTAB extraction, we clearly demonstrated the advantage of using the former. Using our process control strain, we demonstrated that the DNA extraction method was well-suited for wine and especially red wine. Moreover, as PVPP is known to remove PCR inhibitors (Tessonnière et al., 2009) it was added in our assay and improved *C*_*q*_-values. The results of our validation protocol proved the specificity of the assay. Indeed, qPCR primers showed good specificity for all the wine AAB tested and did not amplify from the other wine organisms. Moreover, our study showed that AAB population determination by qPCR with the Lipp method using PVPP was not influenced by the other microorganisms present (*O. oeni* and *B. bruxellensis*) in high concentration. Therefore, pre-amplification using a nested PCR technique (González et al., 2006) is not required to determine the AAB population in samples.

### AAB qPCR limit of quantification

Our LOQ was 5.10^2^ cells with a test sample of 10 ml. This LOQ is similar to those previously reported and was reached with AAB grown in red wines, contrary to González et al. (2006) and Torija et al. (2010). In our study, the correlation coefficient of the standard curve obtained for AAB in red wines was 0.76. This coefficient might appear low but it comes from a calibration curve used for wine. Indeed, it is essential to create calibration curves in food matrices. The creation of standard curves for the quantification of a microorganism by diluting DNA from one extraction, as has been done in most studies, should be avoided (González et al., 2006; Torija et al., 2010; Valera et al., 2013). Consequently, the influence of food matrices is not considered, resulting in an underestimation of microbial load (Cocolin and Rantsiou, 2012). Most studies have obtained a high correlation coefficient between 10-fold serial DNA dilutions, but have not performed DNA extraction from a sample containing a low bacterial population, thus biasing efficiency. DNA extraction must be performed from each dilution before running the qPCR to create a standard curve.

### Evaluation of the method with naturally spoiled wines

Little is known of the spoilage caused by AAB populations and it probably depends on the matrix (sulfites, polyphenols, etc.). Also, this topic is controversial in the literature. According to Joyeux et al. (1984), a low population can activate significant volatile acidity production when exposed to air. However, Drysdale and Fleet (1985) reported that *A. pasteurianus* and *A. aceti* occur at 10^1^–10^3^ CFU/mL in many wines during bulk storage in wineries without causing spoilage. Moreover, Bartowsky and Henschke (2008) have shown that 2.10^4^ *A. pasteurianus*/mL in a Shiraz wine leads to an acetic acid concentration of 0.6 ±0.0 g/L. This wine was not considered spoiled because the initial wine, without microorganisms, contained 0.5 ±0.0 g of acetic acid per liter. Another wine containing 9.10^4^ *A. pasteurianus*/mL had 3.5 ±1.7 g/L of acetic acid, and was thus spoiled. These results may underestimate the true population due to the uncertainty in recovering all the bacteria. In the literature, there is no clear consensus regarding AAB concentration leading to spoilage risk. Therefore, we determined the level of acetic acid concentration in ethanol medium and red wine according to the AAB population over time. In the latter, the LAB concentration was determined to avoid overestimation of the AAB population by FCM bacteria enumeration, even if the LAB is not a serious issue because AAB causes wine spoilage only during aging in the cellar and after bottling (Henick-Kling, 1993), normally without the presence of LAB. In our study, neither yeasts nor LAB were detected in these red wines. Thus, the assessment of our improved qPCR assay for AAB quantification in naturally contaminated red wine proved reliable and efficient in comparison with cytometry results. The small deviations between both methods were probably derived from the matrix, which was different to that used to write the equation. In our study, during the monitoring of AAB naturally present in red wine, red wine exhibiting an AAB population lower than 6 log bacteria/mL was not altered. However, for wines presenting an AAB population of about 6-7 log cells/mL, the acetic acid exceeded the aroma threshold (Davis et al., 1985; Eglinton and Henschke, 1999a,b; Swiegers et al., 2005) but not the European limit values for sale. For the other wines with high AAB populations and during the stationary phase, acetic acid exceeded the European limit values. Thus, the method developed in this study had an elevated LOQ without using a higher sample volume (e.g., 10 mL).

## Conclusion

The method allowed AAB detection and quantification before spoilage occurred, which meets the needs and expectations of the wine industry when monitoring AAB populations on a regular basis. Therefore, in our study, we developed a qPCR method which allows the reliable quantification of AAB in red wine. We showed that the previously reported DNA extraction method was not efficient enough for precise quantification. The methods used in these studies probably led to underestimating the AAB population. Use of an internal control allows validating DNA extraction and qPCR efficiencies. No underestimation can be made if the initial concentration of the internal control added in the sample before DNA extraction is found. Moreover, the standard curve was established with AAB that proliferated in red wine. Finally, as far as we know, this is the first time a qPCR protocol allowing AAB quantification in red wines without bias (neither cell loss nor PCR inhibitor presence) has been validated. Moreover, the presence of other microorganisms in the sample did not alter AAB quantification.

Specific AAB species quantification is not possible with our method; however, our main goal was to quantify AAB in wines whatever the species present in order to evaluate the risk of spoilage. Furthermore, one drawback of the method is that qPCR quantifies live and dead AAB. Propidium monoazide could be used (Vendrame et al., 2013; Rizzotti et al., 2015) but cell exposure to a stress like ethanol, which is known to permeabilize membranes (Alexandre et al., 1994), can result in cells stained by propidium iodide, for example, but that are still alive (Davey and Hexley, 2011). Moreover, Shi et al. (2012) highlighted an underestimation of total yeasts, *S. cerevisiae*, total LAB, non-*O. oeni* LAB, and total AAB in wine by real-time PCR coupled with ethidium monoazide. However, they demonstrated that 40 min of incubation in recovery medium could completely cancel the underestimation of viable cell counts performed in wine, but not cell growth, which must be checked for each sample. To avoid this potential error, one way to circumvent the drawback of the method is to monitor the AAB population over time. Under these conditions, a decrease in *C*_q_-value would reflect AAB growth and potentially the risk of spoilage.

## Author contributions

Conceived and designed the experiments: HA, CL, and MG. Performed the experiments: CL. Generated and analyzed the data: CL, HA, and MG. Wrote the paper: CL, HA, and MG.

## Funding

This work was funded by the Regional Council of Burgundy and the Interprofessional Office of Burgundy Wines.

### Conflict of interest statement

The authors declare that the research was conducted in the absence of any commercial or financial relationships that could be construed as a potential conflict of interest.
